# Supplementary material for: A Global Screen for Assembly State Changes of the Mitotic Proteome by SEC-SWATH-MS
Source: Cell Syst. 2020 Feb 26;10(2):133–155.e6. doi: 10.1016/j.cels.2020.01.001 (PMC7042714; doi:10.1016/j.cels.2020.01.001)

O75506 | HSBP1_HUMAN | HSBP1 HSF1BP
Monomer MW [kDa]: 8.544 Monomer expected elution fraction: 62

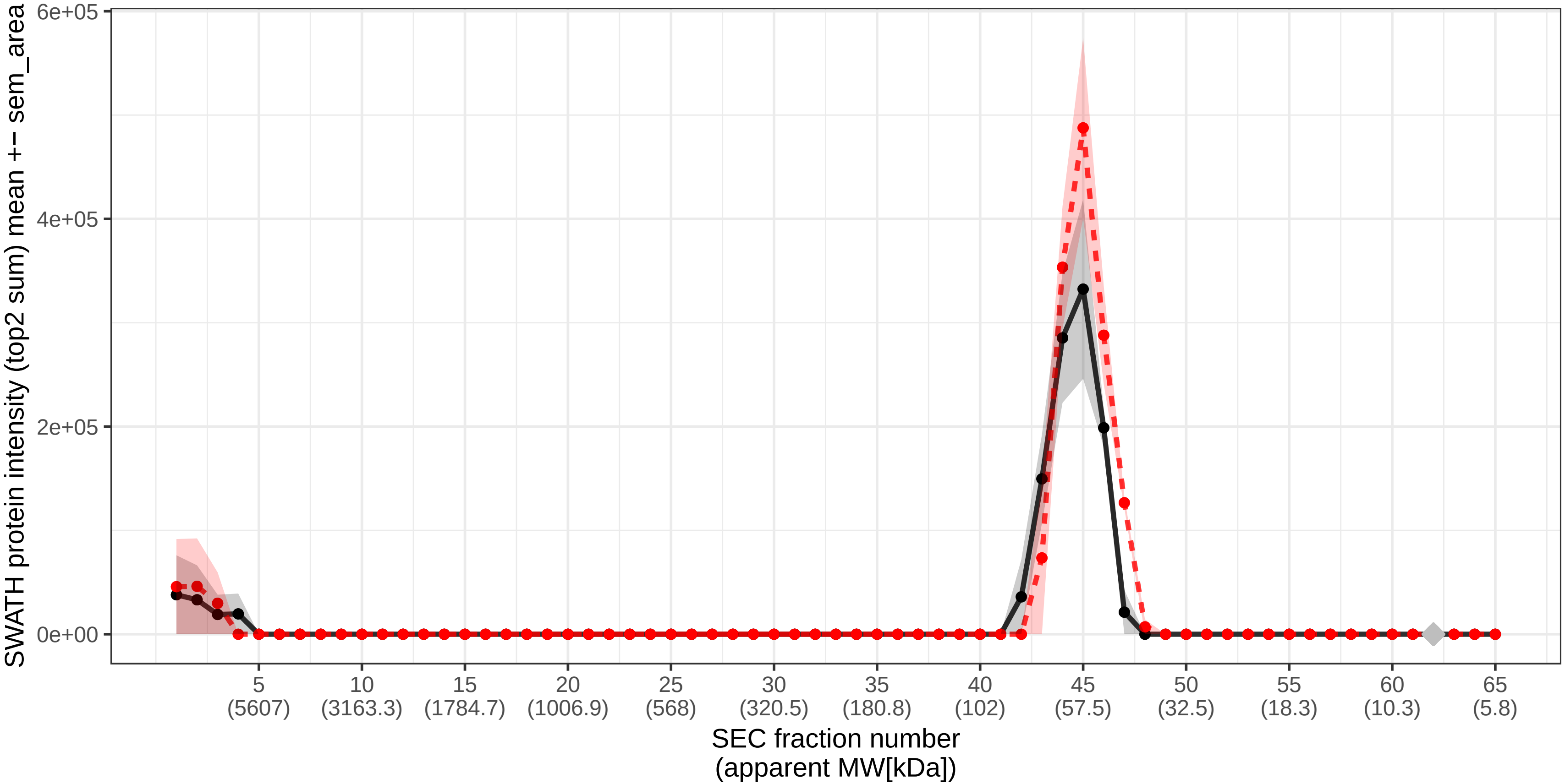

Supplement: Data S1. SEC-SWATH-MS Protein Chromatograms, Related to Figure 1 [file mmc6.zip › SECchrom_O75506_HSBP1_HUMAN_HSBP1_HSF1BP.pdf]
